# Effectiveness of Telemedicine Interventions in Chronic Obstructive Pulmonary Disease (COPD) Management: A Randomized Controlled Trial Comparing Yoga Therapy and Pulmonary Rehabilitation Over Three Months

**DOI:** 10.7759/cureus.56060

**Published:** 2024-03-12

**Authors:** Ruchi Dua, Saloni Malik, Ajeet S Bhadoria, Osama Neyaz, Ajay S Krishnan, Chinmay Pandya

**Affiliations:** 1 Pulmonary Medicine, All India Institute of Medical Sciences, Rishikesh, Rishikesh, IND; 2 Himalayan School of Yoga Sciences, Swami Rama Himalayan University, Dehradun, IND; 3 Community and Family Medicine, All India Institute of Medical Sciences, Rishikesh, Rishikesh, IND; 4 Physical Medicine & Rehabilitation, All India Institute of Medical Sciences, Rishikesh, Rishikesh, IND; 5 Radiation Oncology, Mahamana Pandit Madan Mohan Malviya Cancer Centre, Varanasi, IND; 6 Yoga, Dev Sanskriti Vishwavidyalaya, Haridwar, IND

**Keywords:** yoga therapy, video telemedicine, tele-pulmonary rehabilitation, quality of life, depression, anxiety, dyspnea, exercise capacity, copd

## Abstract

Background

Pulmonary rehabilitation (PR) is an integral part of non-pharmacological therapy in chronic obstructive pulmonary disease (COPD). Yoga therapy (YT) has been shown to be beneficial in COPD, but the lack of large well-designed trials and standardized modules restricts its acceptability. This randomized control trial compares these two modalities in COPD patients via supervised tele-intervention.

Objectives

The primary objective of the study is to compare a 45-minute, five-days-per-week series of tele-YT (T-YT) with tele-PR (T-PR) for three months in terms of exercise capacity (6-Minute Walk Distance (6MWD)) in COPD patients.

Methods

COPD patients were randomly assigned (1:1) to T-YT or T-PR groups in a parallel-arm single-blinded controlled trial. The primary outcome is 6MWD recorded at baseline and after three months and secondary outcomes were symptom scores, Forced expiratory volume in the first second (FEV1), health-related quality of life (HrQoL), and depression and anxiety scores. Assessments were conducted at baseline and at the end of the three-month study period with a sample size of 75 in each group.

Results

A total of 150 consecutive patients with COPD were randomly assigned to either the T-YT (n = 75) or T-PR (n = 75) group. Their mean ± SD ages was 62.5 ± 7.0 years. The T-YT group had 55.5% males and 34.47% females, whereas the T-PR group had 44.5% males and 61.53% females. The trial was completed by 123 patients; 88% in the T-YT group and 76% in the T-PR group. Pre-intervention, the median (range) of 6MWD in T-YT and T-PR groups was 240 (120-600) m and 240 (120-660) m, respectively. There was statistically significant improvement in both groups respectively (p<0.001) post intervention from baseline but no significant intergroup difference (p = 0.486). A similar trend was seen in secondary outcomes with significant intragroup improvements and non-significant inter-group differences except FEV1%, which showed neither intragroup nor intergroup significant improvement.

Conclusion

Using a validated module, a three-month T-YT improves exercise capacity, symptom scores, HrQoL, and depression and anxiety scores similar to T-PR. T-YT is an acceptable alternative to T-PR in the management of COPD.

## Introduction

Chronic obstructive pulmonary disease (COPD) is a systemic inflammatory disorder that limits airflow in the airways and alveoli and etiologies include smoking, biomass fuels, and air pollution [1]. Due to increased longevity and rising air pollution, this major public health issue is projected to increase with a current global prevalence of 10.3%. Ninety percent of deaths among COPD patients aged below 70 years occur in low- and middle-income countries like India [2-4].

Both pharmacological and non-pharmacological treatment strategies are used in the management of COPD. Pulmonary rehabilitation (PR) is a non-pharmacological intervention that involves patient assessment, patient-tailored therapies like exercise training, education, and behavior change to improve physical and mental health [5]. Since the initial European Respiratory Society/American Thoracic Society (ERS/ATS) on PR, the knowledge and acceptance of PR have grown [6-7]. The coronavirus disease 2019 (COVID-19) pandemic and the spread of internet services have sparked research in novel ways of delivering rehabilitation via tele-interventions. Preliminary studies incorporating tele-rehabilitation in COPD patients have shown promising results with improved adherence levels [8].

Yoga therapy (YT) is based on mindful exercises using body, breathing, and mind. It has been tried in diverse disorders with both psychological and physiological benefits being reported. Its beneficial effects have been demonstrated to improve breathing skills, chest expansion, lung function, body posture, and health-related quality of life [9]. Few studies on yoga in COPD have shown benefits but large well-designed randomized control trials (RCTs) comparing YT and PR in COPD are largely lacking. Limited studies on yoga delivered via tele-intervention have proven its acceptability and feasibility in varied disease subsets [10]. In this study, we have compared a three-month tele-YT (T-YT) intervention with a similar tele-PR (T-PR) program in COPD patients. The T-YT module itself has been verified by a pilot trial.

## Materials and methods

This was a single-blinded (statistician and outcome assessor), randomized, parallel-group controlled trial conducted at the outpatient department of a tertiary care center (All India Institute of Medical Sciences (AIIMS), Rishikesh) in India. The trial is registered at ClinicalTrials.gov (CTRI/2020/11/029249) and conforms to the principles of the Helsinki Declaration. The study was approved by the AIIMS Institutional Ethics Committee (approval number: AIIMS/IEC/22/395).

Participant selection

Stable Global Initiative for Obstructive Lung Disease (GOLD) group B-D COPD patients aged 40-75 years, who consented, were included in the study. Patients with congestive heart failure, uncontrolled diabetes mellitus, severe uncontrolled hypertension, chronic liver disease, chronic renal failure, significant co-morbidity limiting acute life expectancy (<3 months), pregnant and lactating mothers, patients in acute exacerbation, and patients unable to perform spirometry, rehabilitation, or yoga for any reason (e.g.-severe visual, auditory or neurological or musculoskeletal involvement) were excluded.

Between June 2021 and April 2022, 250 patients with COPD were screened and 150 patients were recruited as per predefined inclusion and exclusion criteria. Computer-generated block randomization with a block size of 10 (1:1) was used to assign patients to the intervention group (T-YT) and the control group (T-PR) using sealed envelopes.

Sample size calculation

The sample size was calculated using G*Power 3.1 version.

Tests - Means: Difference between two independent means (two groups) Analysis: A priori: Compute required sample size Input: Tail(s) = Two α err prob = 0.05 Power (1-β err prob) = 0.80 Delta = -2.8493 SD = 122.000 Allocation ratio N2/N1 = 1 Output: Sample size group 1 = 59 Sample size group 2 = 59 Total sample size = 118

Assuming a 20% attrition rate, the estimated sample size in each group was 75 and the total sample size was 150.

Study procedure

Both groups received the usual pharmacological treatment as per GOLD guidelines which included beta2 agonists, antimuscarinic drugs, and inhaled corticosteroids as indicated [1]. Apart from this, one group was managed with a 45-minute T-YT session five days a week for three months, while the other group was managed with T-PR. The main goal was to see if T-YT improves 6MWD as much as T-PR. Other factors like breathing difficulty and quality of life were also measured.

T-YT Group (Intervention Group)

T-YT is a combination of asana, pranayama, and kriya and was provided by a certified yoga instructor. Participants were instructed to practice T-YT for 45 minutes daily and sessions were supervised five days/week via Zoom (Zoom Video Communications, Inc., San Jose, California, United States)/Google Meet (Google LLC, Mountain View, California, United States) [11-12].

T-PR Group (Control Group)

T-PR is a combination of exercises, provided by a certified physiotherapist. Participants were instructed to practice T-PR for 45 minutes daily and sessions were supervised five days/week via Zoom/Google Meet [13].

Assessment of outcome measures

Baseline demographics were collected by a structured questionnaire and included age, gender, educational qualification, occupation, income, residence, combined assessment as per GOLD guidelines, and smoking status [1]. The primary outcome was exercise capacity in terms of 6MWD which was performed as per ATS/ERS guidelines [14]. Secondary outcomes included forced expiratory volume in one second (FEV1%), symptom score: COPD assessment test (CAT) and modified medical research council (mMRC) dyspnea scores, health-related quality of life (HrQoL) using St. George Respiratory Questionnaire (SGRQ), depression score using Patient Health Questionnaire (PHQ-9), and anxiety score using Generalized Anxiety Disorder questionnaire (GAD-7). All outcomes were recorded at baseline and end of the third month. Hindi-validated tools were used for CAT, SGRQ, PHQ-9, and GAD-7 questionnaires [15-18].

Statistical analysis

All data was entered into Microsoft Excel (Microsoft Corporation, Redmond, Washington, United States) and analyzed using IBM SPSS Statistics for Windows, Version 23.0 (Released 2015; IBM Corp., Armonk, New York, United States). Continuous variables were reported as mean ± SD. Categorical variables were reported as percentages and analyzed using the chi-square test. Kolmogorov-Smirnov test was used to check for the normality of distribution in each group separately. Paired t-test was used to compare normally distributed variables, baseline vs. post-three months data within each group while Wilcoxon signed ranks test was used to compare non-normally distributed variables, baseline vs. post-three month data within each group. Independent t-test was used to compare similar variables between the two groups' differences if they were normally distributed and Mann-Whitney U test was used to compare similar variables between the two groups' differences if they were non-normally distributed. A value of p<0.05 was considered to indicate a statistically significant result.

## Results

Baseline characteristics

**Figure 1:**
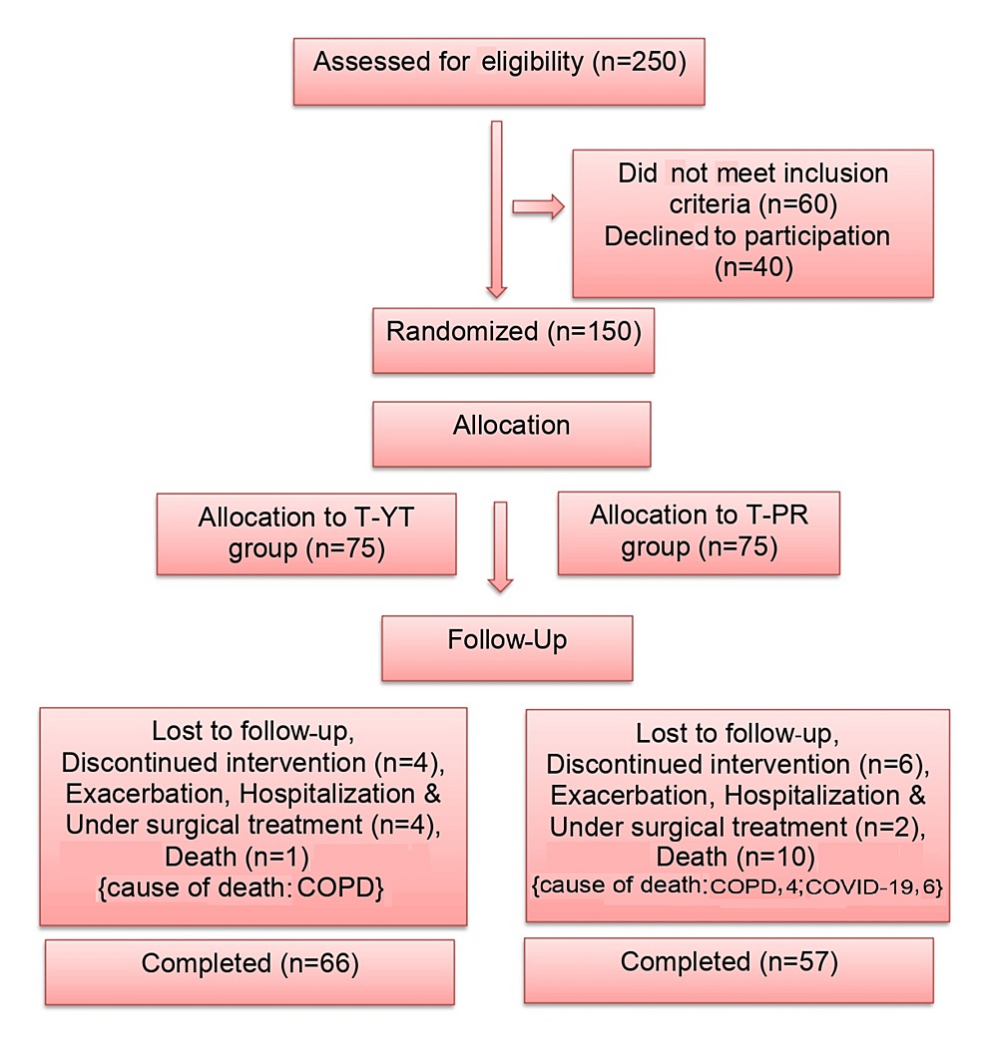
CONSORT Chart CONSORT: Consolidated Standards of Reporting Trials; COPD: chronic obstructive pulmonary disease; T-YT: tele-yoga therapy; T-PR: tele-pulmonary rehabilitation

The progression of participants through the study is depicted in Figure 1. A total of 150 COPD patients were assigned randomly to either the T-YT group (n=75) or the T-PR group (n=75).

Table 1 shows the comparison of baseline characteristics between both groups. The average age (mean± SD) in the T-YT group was 62.1 ± 6.8 years and that in the T-PR group was 63.0 ± 7.3 years (p = 0.87). Of the patients in the T-YT group, 92.4% were males and the remaining 7.5% were females while 86% of patients in the T-PR group were males and the remaining 14% were females. At baseline, FEV1% for the T-YT group was 61.2 ± 25.2% and for the T-PR group, it was 61.8 ± 26.2%. In both groups, the majority of patients were literate, with 63.64% and 71.93% having completed up to middle school in the T-YT group and T-PR group, respectively. In the T-YT group, 39.40% belonged to rural areas while 19.3% belonged to rural areas in the T-PR group. A monthly income of less than Rupees 10,000 was seen in 93.94% and 91% in the T-YT group and T-PR arm, respectively. In the T-YT group, group assessment for group B comprised 53.04%, group C comprised 22.73%, and group D comprised 24.25% of the patients, and in the T-PR group, group assessment for group B comprised 56.15%, group C comprised 33.34%, and group D comprised 10.53% of the patients.

**Table 1:** Comparison of baseline characteristics of categorical variables between two groups P value of <0.05 is considered significant, The data has been represented as n (%).

Baseline characteristics		Tele-Yoga Therapy Group (Intervention) (N=66)	Tele-Pulmonary Rehabilitation Group (Control) (N-57)	p-value
		Frequency (Percentage)	Frequency (Percentage)	
Age (years)	41-45	1 (1.50)	0 (0)	0.8781
	46-50	3 (4.54)	3 (5.27)	
	51-55	11 (16.70)	9 (15.79)	
	56-60	11 (16.70)	7 (5.70)	
	61-65	15 (22.73)	11 (19.30)	
	66-70	24 (36.37)	25 (43.86)	
	71-75	1 (1.52)	2 (3.51)	
Gender	Male	61 (92.43)	49 (85.97)	0.2453
	Female	5 (7.58)	8 (14.04)	
Educational Qualification	Up to middle school	42 (63.64)	41 (71.93)	0.6854
	Up to intermediate school	19 (28.79)	13 (22.81)	
	Graduation	2 (3.04)	2 (3.51)	
	Post-Graduation	3 (4.55)	1 (1.76)	
Occupation	Farmer	57 (86.37)	46 (80.71)	0.7556
	Government service	2 (3.04)	4 (7.02)	
	Business	3 (4.55)	3 (5.27)	
	Homemaker	4 (6.07)	4 (7.02)	
Income	1000-10000	62 (93.94)	52 (91.23)	0.7577
	11000-30000	3 (4.55)	3 (5.27)	
	31000-50000	1 (1.52)	2 (3.51)	
Residence	Rural	26 (39.40)	11 (19.3)	0.0154
	Urban	40 (60.61)	46 (80.71)	
Combined Assessment	B	35 (53.04)	32 (56.15)	0.1046
	C	15 (22.73)	19 (33.34)	
	D	16 (24.25)	6 (10.53)	
Smoking Status	Smoker	16 (24.25)	14 (24.57)	0.0401
	Ex-Smoker	46 (69.70)	31 (54.39)	
	Non-Smoker	4 (6.07)	12 (21.06)	

Though both groups were comparable in terms of age, gender, spirometric severity, combined assessment distribution, baseline 6MWD, mMRC, CAT, SGRQ, PHQ-9, and GAD-7 scores, more patients in the T-YT group are from rural areas and contained a greater proportion of ex-smokers Table 1. The 3-month follow-up assessments were completed by 123 patients (66/75 (88%) in the T-YT group and 57/75 (76%) in the T-PR group).

Comparison of change in exercise capacity (6MWD) over three months

6WMD indicates the patient's exercise capacity and also has prognostic implications. Pre-intervention, the median 6MWD in T-YT and T-PR groups was 240 (range: 120-600) m and 240 (range:120-660) m, respectively, and the change shown by a median of T-YT and T-PR was 165 (range: (-270)-540) m and 120 (range: (-180)-450) m. Wilcoxon test within the group revealed statistically significant improvement in both groups (p<0.001). However, the independent t-test revealed no statistically significant difference between the groups (p=0.486). COPD patients in both the T-YT and T-PR groups showed improvement, which was not statistically significantly different, between the two groups.

Comparison and change in secondary outcomes in both groups over three months

CAT And mMRC Scale Scores

At baseline, the mean ± SD of CAT scores in T-YT and T-PR groups was 15.3±6.3 and 16.1±6.3, respectively. The paired t-test showed statistically significant improvement (p<0.001) within both the groups at the end of three months. However, the independent t-test found no statistically significant difference between the two groups (p=0.746). At baseline, the median mMRC scores in T-YT and T-PR groups was 3 (range, 1-4). The Wilcoxon Signed Ranks test was performed within each group, and both groups showed statistically significant intra-group improvement (p<0.001). Post intervention, COPD patients in both the T-YT and the T-PR groups improved in terms of CAT scores and mMRC with no significant inter-group difference.

HrQoL Score 

The SGRQ Scale reveals the health impairment or quality of life of COPD patients. Pre-intervention, the mean ± SD of SGRQ scores in T-YT was 49.5 ± 14.2, and the median (range) of the T-PR group was 53.33 (13.33-73.64). Paired t-test revealed a statistically significant improvement in the T-YT group (p<0.001) and the Wilcoxon signed-ranks test revealed a statistically significant improvement in the T-PR group (p<0.001). The independent t-test, on the other hand, revealed no statistically significant difference between the two groups (p=0.669).

Depression Score

Baseline mean ± SD of PHQ-9 scores in T-YT and T-PR were 10.7±4.8 and 11.1±4.8, respectively. Paired t-test was performed pre and post intervention in the two groups, revealing a statistically significant improvement (p<0.001). In contrast, changes in the mean ± SD were (-5.23) ± 4.08 and (-5.46) ± 4.32 in the T-YT group and T-PR group, respectively. The independent t-test revealed no statistically significant difference between the two groups (p=0.764).

Anxiety Score

Baseline mean ± SD of GAD-7 scores in T-YT and T-PR groups were 8.6±3.7 and 8.0±3.8, respectively. A paired t-test revealed a statistically significant improvement (p<0.001) within the two groups. Change in GAD-7 by mean ± SD was (-4.13) ± 3.22 and (-3.69) ± 3.24 in the T-YT group and T-PR group, respectively. The independent t-test, in contrast, found no statistically significant difference between the two groups (p=0.455).

Lung Function

Baseline mean±SD of FEV1% T-YT and T-PR were 61.2±25.2 and 61.8±26.3, respectively. A paired t-test revealed that neither T-YT (p-0.289) nor T-PR (p=0.328) demonstrated statistically significant improvement post-intervention. The independent t-test revealed no statistically significant difference between the two groups (p=0.977) with the post-intervention mean ± SD of T-YT being 4.32 ± 32.80 and that of T-PR being 4.50 ± 34.40.

## Discussion

PR is a key component of non-pharmacological management in COPD with studies utilizing both onsite to online modes of delivery, especially during the COVID-19 pandemic. In a single-blinded, multicentric, superiority RCT, 134 participants were assigned 1:1 to 10 weeks of 60-minute group-based pulmonary tele-rehabilitation (PTR) three times weekly or conventional PR (90 minutes, two times weekly). The primary outcome was the change in 6MWD from baseline to 10 weeks. The analysis showed no between-group differences for changes in 6MWD after the intervention (9.2 m (95%CI: −6.6 to 24.9)) or at the 22-week follow-up (−5.3 meters (95%CI: −28.9 to 18.3)). More participants completed the PTR intervention (n=57) than conventional PR (n=43) (χ2 test p<0.01) [18].

A 2021 systematic review found that tele-rehabilitation for chronic respiratory illnesses yielded comparable results to center-based PR without safety issues [19]. It included a total of 15 studies (32 reports) with 1904 participants, using five different models of tele-rehabilitation and 99% of study participants had COPD. Tele-rehabilitation had a 93% completion rate (95%CI: 90-96%) compared to 70% for in-person rehabilitation and no detrimental effects compared to in-person or no therapy. Other studies comparing T-PR to center-based rehabilitation in COPD patients showed no difference in 6MWT distance (p=0.563), mMRC (p=0.911), and CAT (p=0.85) and concluded tele-rehabilitation is effective in improving exercise tolerance and patient-reported outcomes and a valid alternative to center-based rehabilitation [20]. Similar to these studies, we used T-PR in the control arm delivered by trained physiotherapists via Google Meet/Zoom and found a significant improvement in 6MWD, mMRC, and CAT scores in the T-PR group.

While large-scale research using standardized protocols is sparse, existing yoga trials in COPD offer promising potential. A pilot study with 43 patients compared 12 weeks of pranayama breathing exercises combined with education against education alone [21]. The pranayama group saw a 28 m increase in 6MWD compared to a 15 m decrease in the control group (borderline statistically significant). Pranayama also led to small increases in inspiratory capacity and reduced air trapping while both groups experienced improvements in symptom measures without significant differences in overall lung function or inflammatory markers. Yoga, particularly pranayama, shows promise in improving exercise capacity and potentially lung function in COPD patients. Larger, standardized trials are crucial to solidify these findings and explore the mechanisms behind yoga's benefits.

In another study, the yoga group showed a significant increase in FEV1, 6MWD, and quality of life using the SGRQ questionnaire after 12 weeks of yoga (p < 0.05) when compared with the control group [22]. In a study among COPD patients who practised selected yoga exercises including breathing exercises, meditation, and yoga postures for one hour, thrice a week, for six weeks under a certified yoga therapist, HrQoL and lung function were assessed at baseline and the end of six weeks [23]. Statistically significant improvements (P < 0.05) were observed for SGRQ, vital capacity, maximal inspiratory pressure, and maximal expiratory pressure. The authors concluded that yoga when practiced by patients with COPD resulted in improvement in HrQoL and lung function on a short-term basis.

In a pilot study, T-YT for heart failure in COPD patients was compared to educational materials, which include information leaflets mailed to participants with one weekly phone call. While small, the study suggested positive effects [10]. Patients enjoyed the home-based format, reported symptom improvement, and felt less isolated. Though technical issues arose, T-YT's potential benefits seem linked to the combination of physical postures, breathing exercises, and relaxation techniques. Similarly, in the current study, significant intragroup improvement was seen in the T-YT group in 6MWD, SGRQ scores, and symptom scores like mMRC and CAT scores but not FEV1%.

In a systematic review and meta-analysis on the effectiveness of yoga training in COPD patients, which included 10 studies, patients performing yoga were reported to have substantial improvement in 6MWD, Borg scale scores, FEV1%, partial pressure of arterial carbon dioxide (PaCO2), SGRQ, and CAT; however, there were no discernible variations in FEV1% or other spirometric measurements [24]. On the other hand, T-YT did not significantly enhance FEV1% in the current trial; however, significant improvements were seen in 6MWD, CAT, mMRC, and SGRQ scores.

A study that performed an online survey among patients and healthcare professionals, including pulmonary care physicians, rehabilitation nurses, and physical and respiratory therapists in India who have been using and practicing smartphone-based tele-rehabilitation, found that while 71% (n = 37/52) of healthcare professionals surveyed were aware of smartphone-based tele-rehabilitation, implementation was extremely low (10%) [25]. Though the majority of COPD patients (n = 21/30; 70%) agreed to accept smartphone-based tele-rehabilitation as one of their treatment options, challenges to efficient implementation included a lack of infrastructure, perceived time consumption, a lack of expertise/training, organizational support, and perceived inefficacy. In the current study, high completion rates were seen in both groups (88% in T-YT and 76% in T-PR).

There are many studies that have demonstrated a significant level of anxiety and depression among obstructive airway disease patients [26,27]. PR shows short-term benefits for anxiety and dyspnea in COPD, but long-term effects need larger studies. Yoga reduces depression and anxiety in various diseases, with limited evidence for anxiety reduction in COPD, especially in elderly and long-duration patients [28]. Similar to these results, in the current study too, both T-PR and T-YT groups had improved PHQ9 and GAD7 scores at the three-month follow-up but without significant inter-group difference. To the best of our knowledge, there is only one published abstract directly comparing PR and YT [29]. The only other study is the pilot validation study of the authors of the current study [11].

This trial is a novel attempt to compare a validated yoga module with a PR module in patients of COPD with both interventions delivered via tele-mode. It proves no significant difference between the T-YT group and T-PR group in terms of improvement in 6MWD, CAT, mMRC, SGRQ, PHQ-9, and GAD-7 scores. High completion rates in both groups highlight the increasing acceptability of tele-intervention in COPD. It also aids in establishing the safety and benefits of T-YT in COPD patients. YT is more culturally appropriate and may have higher chances of adherence, as is seen in the current study, placing it as a viable adjunct/alternative to T-PR among COPD patients.

The study has a few limitations. This was a tele-intervention and an offline study is needed. Also, intervention time could be more than three months.

## Conclusions

Using a validated T-YT module, the study demonstrated that a three-month T-YT is comparable to a T-PR in terms of exercise capacity, symptom scores, SGRQ, PHQ-9, GAD-7, and HRV. T-YT has the potential to be used as a non-pharmacological adjunct treatment option for COPD patients apart from T-PR due to the fact that it is simple to use, has a low cost, does not have any potential bad effects, and is culturally acceptable.
